# Supplementary material for: Frictional Damping from Biomimetic Scales
Source: Sci Rep. 2019 Oct 10;9:14628. doi: 10.1038/s41598-019-50944-0 (PMC6787062; doi:10.1038/s41598-019-50944-0)
Supplement: Supplementary file 1 — Supplementary information [file 41598_2019_50944_MOESM1_ESM.pdf]

Frictional Damping from Biomimetic Scales

Hessein Ali, Hossein Ebrahimi, Ranajay Ghosh*

Department of Mechanical and Aerospace Engineering, University of Central Florida, Orlando FL

Email:ranajay.ghosh@ucf.edu

1. Mass of scales effect

In order to see the influence of scales mass as compared to the substrate, we look at the mass ratio of the two. Assuming a unit width for both mass and scales, one can write the mass of the beam as $M_B = \rho_B h L_B$ while the mass of scales is $M_s = N_s \rho_s (L + l) D$. Their ratio can then be written as $M_r = \frac{M_B}{M_s} = \frac{\rho_B d h}{\rho_s (L+l) D}$ based on uniform distribution of scales (i.e. $L_B = N_s d$) with N_s being the total number of scales on the beam. Knowing that $\eta = \frac{l}{d}$, the mass ratio can be further simplified to $M_r = \frac{\rho_B}{\rho_s} \frac{\frac{h}{L}(L/D)}{\eta(1+\frac{L}{l})}$. In the limits $\frac{L}{D} > 3$, $\frac{L}{h} < 0.1$ and $\frac{L}{l} < 0.1$, the mass ratio is approximately $\frac{M_B}{M_s} \approx \frac{\rho_B}{\rho_s}\left(\frac{30}{\eta}\right)$. Note that these limits are ideal for various types of scales located in nature [1]. Additionally, in the case of small η, the mass of the beam becomes dominant which allows for neglecting the mass of scales. This justifies our assumption of neglecting the kinetic energy of the scales when deriving the EOM derived in the next section.

2. Derivation of Equation of Motion (EOM) of Scaly Beam

To derive the equation of motion, we employ Hamilton principle which requires the need of formulating the kinetic and potential energy of our scaly beam. We can write the strain energy per unit length $\hat{V}_B$ for the entire substrate as $\hat{V}_B = \frac{1}{2} E_B I_B \tilde{\kappa}^2$ with I_B being the second moment of area of the cross section of the beam, $\tilde{\kappa} = \frac{\partial^2 \tilde{y}}{\partial \tilde{x}^2}$ is the instantaneous curvature of substrate, and $\tilde{y}$ is the transverse deflection of the beam, positive upward and negative downward, **Fig.(1)**. To formulate the strain energy brought about scales rotation, we assume a density function for scale RVE distribution, $\lambda(\tilde{x})$ which makes the total scales be $N_{RVE} = \int_0^{L_B} \lambda(\tilde{x}) dx$. In addition, this works addresses uniformly distributed scales along the length of the beam (i.e. $\lambda = \frac{N_{RVE}}{L_B}$). Therefore, the strain energy induced by scales rotation is $\hat{V}_s = \sum_{RVE} \frac{1}{2 \mathrm{N_{RVE}} \tilde{d}} \tilde{K}_s (\theta_{RVE} - \theta_0)^2$. Here the term θ_{RVE} is simplified using Taylor series to $\theta_{RVE} \approx$

$\left(\eta - \frac{1}{2}\right)\psi_{RVE} + \left(\frac{\eta^3}{6} - \frac{\eta}{8}\right)\psi_{RVE}^3 + (\frac{\eta}{384} - \frac{\eta^3}{16} + \frac{3\eta^5}{40})\psi_{RVE}^5$. The dissipation energy density due to interfacial friction is $\hat{V}_d = \sum_{RVE} \frac{1}{N_{RVE}\tilde{d}} \int_{\tilde{k}_e}^{\tilde{k}} f_{fr}\,\mathrm{sgn}(\dot{\tilde{y}}_{RVE})\, d_r$, where $\tilde{\kappa}_e$ is the substrate curvature at the instant $\theta_{RVE} = \theta_0$ and f_{fr} is the frictional force for each RVE and can be derived from **Fig.(1)** as $f_{fr} = \frac{\sin(\beta)(\theta_{RVE}-\theta_0)}{\cos(\psi_{RVE}+\beta)-\bar{r}\cos(\beta)}$ [2]. Here the friction angle $\beta = \tan^{-1}\mu$ and $\bar{r} = \frac{\sin\left(\theta_{RVE}-\frac{\psi_{RVE}}{2}\right)}{\sin\left(\theta_{RVE}+\frac{\psi_{RVE}}{2}\right)}$ [2]. Moving to the kinetic energy, we express the kinetic energy of the substrate as $\hat{T}_B = \frac{1}{2}\rho_B A\left(\frac{\partial\tilde{y}}{\partial\tilde{t}}\right)^2$, where A is again the cross sectional area of the substrate. The work done by the applied load in this work is evaluated through the formula $W = \int_0^L f(x,t)y(x,t)$.

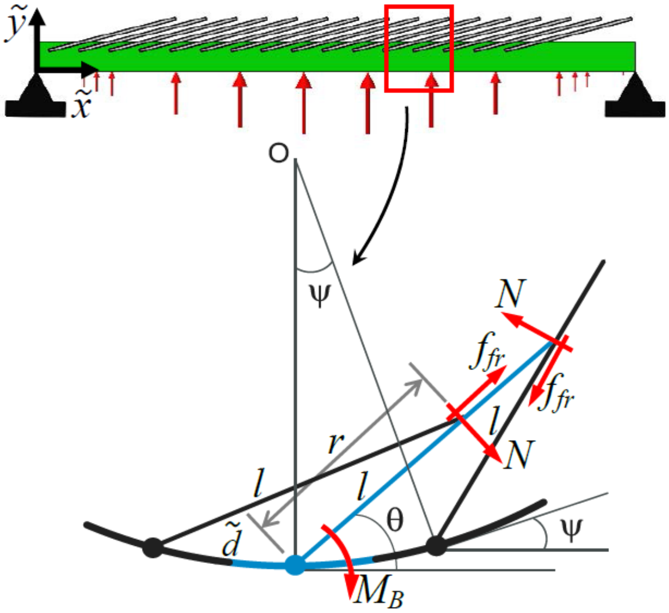

Figure 1: The RVE geometry results due to the assumption of local periodicity where all neighboring scales are placed on a substrate that deform uniformly.

Plugging in all the energy terms with the work done in the Hamilton principle $\delta\int_{t_1}^{t_2}(\hat{T} - \hat{V} + W)dt = 0$ yields the following partial differential equation

$$\rho_B A \frac{\partial^2 \tilde{y}}{\partial \tilde{t}^2} + E_B I_B \frac{\partial^4 \tilde{y}}{\partial \tilde{x}^4} + \tilde{C}\frac{\partial \tilde{y}}{\partial \tilde{t}} + \frac{\partial^2}{\partial \tilde{x}^2}\frac{1}{N_{RVE}}\left[\sum_{RVE} \widetilde{K}_s(\theta_{RVE} - \theta_0)\frac{\partial \theta_{RVE}}{\partial \psi_{RVE}} + \sum_{RVE} \frac{\sin(\beta)\, \widetilde{K}_s(\theta_{RVE} - \theta_0)\, \mathrm{sgn}(\dot{\tilde{y}})}{\cos(\psi_{RVE} + \beta) - \bar{r}\cos(\beta)}\right] H(\tilde{\kappa}_{RVE} - \tilde{\kappa}_e) = \tilde{f}(\tilde{x}, \tilde{t}) \quad (1)$$

After nondimensionalizing the above EOM, we obtain a second order ordinary differential equation using Bubnov-Galerkin weighted residual method. Mathematically it is found through evaluating the following integral $\int_0^1 (EOM)\phi(x)dx = 0$ [3]. Note that $\phi(x) = sin(\pi x)$ for the case of simply supported beam.

Lastly, the massive functions f_1 and f_2 in the nonlinear differential equation are evaluated as

$$f_1 = \frac{\partial^2}{\partial x^2}\int_0^1 \frac{1}{\gamma}\sum_{RVE} K_s(\theta_{RVE} - \theta_0)\frac{\partial \theta_{RVE}}{\partial \psi_{RVE}}\sin(\pi x).$$

With $\psi_{RVE} = dT\frac{\partial^2}{\partial x^2}(\sin(\pi x))$

Similarly

$$f_2 = \frac{\partial^2}{\partial x^2}\int_0^1 \frac{1}{\gamma}\sum_{RVE} \frac{\sin(\beta)\, K_s(\theta_{RVE} - \theta_0)\, \mathrm{sgn}(\dot{\tilde{y}})}{\cos(\psi_{RVE} + \beta) - \bar{r}\cos(\beta)}\sin(\pi x).$$

REFERENCES

1. Browning, A., C. Ortiz, and M.C. Boyce, *Mechanics of composite elasmoid fish scale assemblies and their bioinspired analogues.* Journal of the mechanical behavior of biomedical materials, 2013. **19**: p. 75-86.
2. Ghosh, R., H. Ebrahimi, and A. Vaziri, *Frictional effects in biomimetic scales engagement.* EPL (Europhysics Letters), 2016. **113**(3): p. 34003.
3. Rao, S.S., *Vibration of continuous systems*. 2007: John Wiley & Sons.
